# Regional analgesia using ultrasound-guided intermediate cervical plexus block versus cervical erector spinae block for anterior cervical spine surgery: a randomized trial

**DOI:** 10.1186/s12871-024-02533-6

**Published:** 2024-04-22

**Authors:** Alshaimaa Abdel Fattah Kamel, Ahmed M. Fahmy, Heba M. Fathi, Wael Abd Elrahman Ali Elmesallamy, Osama Yehia A. Khalifa

**Affiliations:** 1 Anaesthesia, Intensive Care and Pain Management Department, Faculty of Human Medicine, Zagazig University, Alsharkia, Egypt; 2 Anesthesia, Intensive Care and Pain Management Department, Faculty of Medicine, Zagazig University, Algamaa Street, Alsharkia, Egypt; 3 Neurosurgery, Faculty of Medicine, Zagazig University, Alsharkia, Egypt

**Keywords:** Regional analgesia, Intermediate cervical plexus block, Cervical erector spinae block, Anterior cervical spine surgery

## Abstract

**Background:**

Regional analgesia techniques are crucial for pain management after cervical spine surgeries. Anesthesiologists strive to select the most effective and least hazardous regional analgesia technique for the cervical region. Our hypothesis is that an intermediate cervical plexus (IC) block can provide adequate postoperative analgesia compared to a cervical erector spinae (ES) block in patients undergoing anterior cervical spine surgery.

**Methods:**

In this double-blind prospective trial, 58 patients were randomly assigned into two equal groups prior to the administration of general anesthesia. Patients in the IC group (*n* = 29) underwent ultrasound-guided bilateral intermediate cervical plexus block with 15 ml of bupivacaine 0.25% administered to each side. The ES group (*n* = 29) underwent ultrasound-guided bilateral cervical erector spinae plane blocks with 15 ml of 0.25% bupivacaine administered to each side at the C6 level. The primary outcome was to record the time to the first call for rescue analgesia (nalbuphine), and the secondary outcomes were to measure the performance time, the onset of the sensory block, the intraoperative fentanyl consumption, postoperative pain intensity using VAS, the postoperative total nalbuphine consumption, and postoperative complications such as nausea, vomiting, hypotension, and bradycardia.

**Results:**

The performance and onset of sensory block times were significantly shorter in the IC group compared to the ES group. The time to first call for nalbuphine was significantly shorter in the IC group (7.31 ± 1.34 h) compared to the ES group (11.10 ± 1.82 h). The mean postoperative VAS scores were comparable between the two groups at the measured time points, except at 8 h, where it was significantly higher in the IC group, and at 12 h, where it was significantly higher in the ES group. The total nalbuphine consumption was significantly higher in the IC group (33.1 ± 10.13 mg) compared to the ES group (22.76 ± 8.62 mg).

**Conclusions:**

For patients undergoing anterior cervical spine surgery, the intermediate cervical plexus block does not provide better postoperative regional analgesia compared to the cervical erector spinae block. Performance time and onset time were shorter in the IC group, whereas nalbuphine consumption was lower in the ES group.

**Trial registration:**

The trial was registered at clinicaltrials.gov. (NCT05577559, and the date of registration: 13–10-2022).

## Background

Anterior cervical spine surgery is a common procedure for treating disc herniation, cervical spondylosis, and spinal cord vascular diseases and tumors [1]. Surgeons have been able to effectively treat patients with various cervical spine conditions by using specialized retractors, natural muscle planes, and paying careful attention to surrounding structures [2]. However, despite the high success rates associated with these surgeries, patients often experience postoperative pain between the shoulder blades and in the neck. Improper perioperative pain management after anterior cervical spine surgery can impact a patient's recovery, overall health, and ability to swallow, and may lead to nausea, vomiting, and chronic pain [3].

A regional analgesia block is a technique used to suppress nerve transmission and alleviate or prevent pain. It is commonly used in combination with general anesthesia or as the sole anesthetic technique, particularly in orthopedic, plastic and vascular surgeries to reduce the amount of anesthetic and analgesic agents needed, minimize systemic side effects, improve recovery, provide better postoperative pain relief, and shorten hospital stays [4].

The cervical plexus is made up of the ventral rami of the C1-C4 spinal nerves. The cervical plexus consists of motor (phrenic nerve, direct muscle branches) and terminal sensory branches (C2-C4): the supraclavicular nerves, the lesser occipital nerve, greater auricular nerve, and transverse cervical nerve. The latter is formed in the compartment between the prevertebral and superficial layer of the cervical fascia, deep to the SCM. [5]. Before 2004, any cervical plexus blocks that were performed superficially to the prevertebral fascia were termed superficial cervical plexus blocks. However, Telford and Stoneham suggested the term intermediate cervical plexus block to distinguish between the superficial block (subcutaneous or subplatysmal) and the block in the interfascial compartment [6]. Intermediate cervical plexus block is a safe, popular, easy, and effective technique for regional analgesia of cervical region [7]. The use of ultrasound guidance reduces the risk of complications, allows for real-time visualization of anatomical structures, and helps guide needle placement [8].

Bilateral cervical erector spinae block using ultrasound has recently been studied for shoulder surgery [9]. Since the brachial plexus, phrenic nerves, cervical nerve roots, and deep cervical muscles are enclosed in the prevertebral fascia, injecting a local anesthetic near the cervical transverse process (TP) can spread to nearby structures within the prevertebral compartment. In a cadaver study, it was found that injecting a 20 mL dye solution into the TP of C6 or C7 resulted in staining of the C5-T1 nerve roots [10].

With the increasing number of nerve block techniques available, anesthesiologists may have difficulty determining the most appropriate technique to achieve optimal recovery after anterior cervical spine surgery. Therefore, this study was conducted to compare the analgesic effects of ultrasound-guided intermediate cervical plexus block and cervical erector spinae block in patients undergoing anterior cervical spine surgery.

## Methods

### Study design

This study was conducted in accordance with the regulations and guidelines of Helsinki. It was approved by our university's Institutional Review Board (IRB#9790, 4–10-2022) and registered at Clinicaltrials.gov (NCT05577559, 13–10-2022). The first patient was enrolled on November 1st. Written informed consent was obtained from all patients.

This randomized prospective clinical study was conducted on 58 patients scheduled for anterior cervical spine surgery under general anesthesia from November 1st, 2022, to December 2023. Patients of both sexes, aged 21 to 60 years old, with American Society of Anesthesiologist Physical Status (ASA PS) I and II, and a body mass index (BMI) from 25 to 35 kg/m2 were included in the study. However, patients with a local infection at the puncture site, altered mental status, a history of allergy to study drugs (bupivacaine, fentanyl), hematological disorders including coagulation abnormalities, severe hepatic or kidney impairment, or chronic pain, were excluded from the study.

The patient has the right to withdraw from the study at any time without any negative consequences for their medical or surgical treatment plan.

The primary outcome was to record the time to the first call for rescue analgesia (nalbuphine), and the secondary outcomes were to measure the performance time, the onset of the sensory block, the intraoperative fentanyl consumption, postoperative pain intensity using VAS, the postoperative total nalbuphine consumption, and postoperative complications such as nausea, vomiting, hypotension, and bradycardia.

After a routine pre-operative evaluation, surgery was performed under general anesthesia. For all patients, an intravenous (IV) line was inserted and IV fluid was started. Monitors were attached to the patients to record basal vital data, including respiratory rate, oxygen saturation, heart rate, and blood pressure. Before the induction of general anesthesia, the patients were divided into two groups using computer-generated randomization table:

*IC group (n* = *29)*: Patients received a bilateral ultrasound-guided intermediate cervical plexus block using 15 ml of bupivacaine 0.25% for each side.

*ES group (n* = *29)*: Patients received a bilateral ultrasound-guided cervical erector spinae plane block using 15 ml of bupivacaine 0.25% for each side at the level of C6.

### Block technique

All blocks were conducted under sterile conditions in the operating room with sedation “midazolam 0.03–0.05 mg/kg as needed”.

#### Materials

- Ultrasound Machine: a portable ultrasound system (MTurbo; FUJIFILM Sonosite Inc., Bothwell, Washington, USA)
- Probe: A linear array transducer (6–13 MHz frequency).
- Needle: 22-gauge spinal needle, length (70 mm in superficial cervical block and 88 mm in cervical erector spinae block).

##### Ultrasound guided intermediate cervical plexus block [11]

**Fig. 1:**
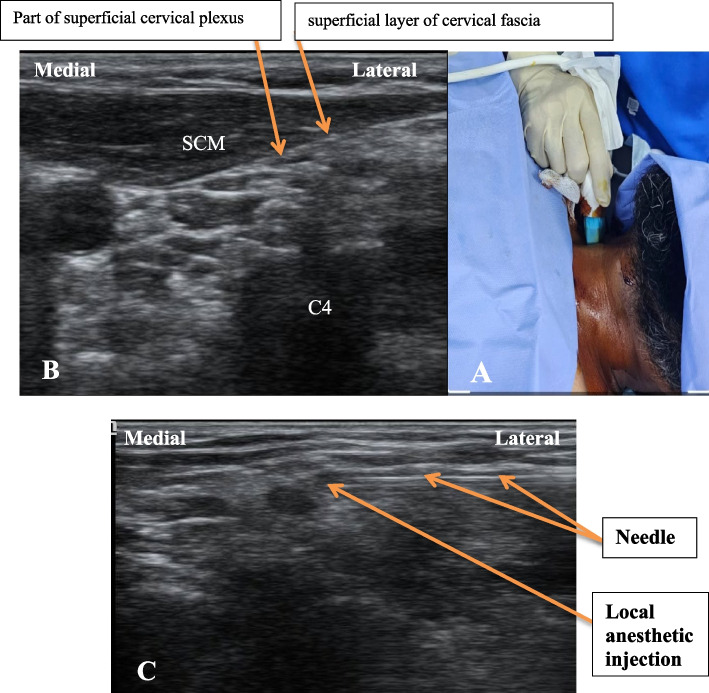
**A** Site of ultrasound probe. **B** Sono-anatomy of Superficial cervical block. **C** Local anesthetic injection

The patient was positioned supine with a slight elevation of the head and the head turned away from the blocked side. After sterilizing the skin, the ultrasound probe was placed over the middle of the posterior border of the sternocleidomastoid muscle (Fig. 1A and B). The needle was inserted in-plane while keeping the probe in a transverse position. The needle tip was placed under the sternocleidomastoid muscle and below the superficial fascia, and then 15 ml of 0.25% bupivacaine was injected (Fig. 1C). The spread of the local anesthetic was visualized using ultrasound guidance. The same steps were repeated on the other side.

##### Ultrasound guided bilateral cervical Erector spinae block [12]

**Fig. 2:**
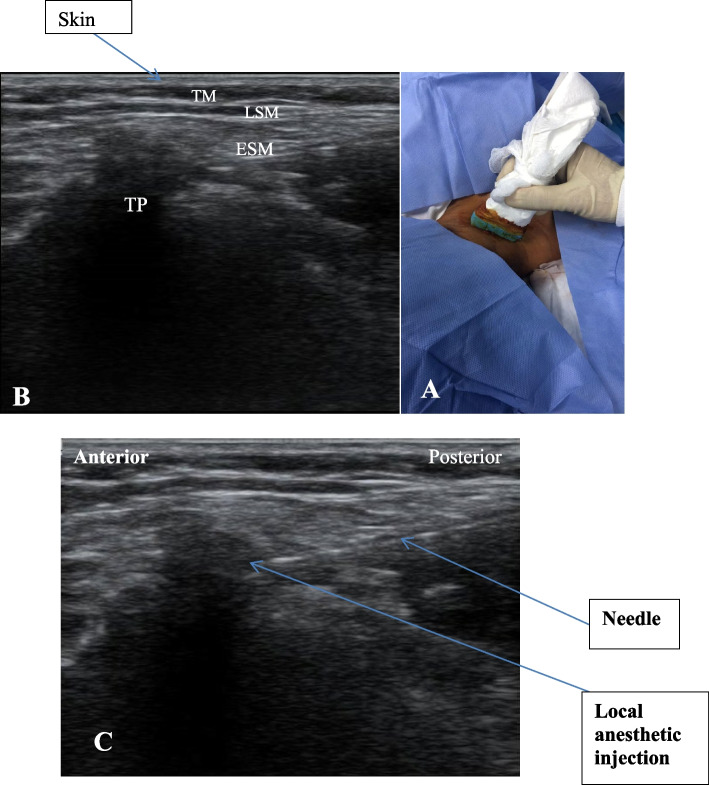
**A** Site of ultrasound probe insertion. **B** Sono-anatomy of cervical erector spinae block (TM = Trapezius muscle, LSM = Levator scapula muscle, ESM = erector spinae muscle and TP = transverse process). **C** Local anesthetic injection

The patient was positioned laterally, with a pillow under their head. Transverse ultrasound scanning of the lower cervical area was done. Starting from the supraclavicular brachial plexus, the transducer was slid in a cephalic direction to show the transverse process of the C7 vertebra. Then, it was moved further cephalic to display the transverse process of C6, along with its characteristic anterior and posterior tubercles. The transducer was then slid posteriorly to show the posterior tubercle of C6, along with the posterior neck muscles above it (trapezius, levator scapula, and erector spinae) (Fig. 2A and B). After prepping and draping the patient using aseptic technique, the needle was inserted (in plane technique from posterior) until it reached the posterior tubercle of C6. Then, 15 ml of bupivacaine 0.25% was injected. The same steps were repeated on the opposite side (Fig. 2C). The patient's ability to detect pinprick sensation was assessed every 5 min for 30 min following the block.

General anesthesia was induced 30 min after the block using fentanyl (1.5 ug/kg iv), propofol (2 mg/kg iv) and atracurium (0.5 mg/kg iv), and maintained after orotracheal intubation as balanced anesthesia using isoflurane ( 1 MAC). Fentanyl 0.5 ug/kg was administered based on the heart rate and mean arterial blood pressure of patients, if it increased by more than 20% from the baseline measurement after excluding other causes. Mechanical ventilation was adjusted to maintain ETCO2 (end tidal CO2) at 35 to 40 mmHg.

At the end of the surgery, the inhalational anesthetic was turned off and the muscle relaxant was reversed using a combination of neostigmine 0.05 mg/kg and atropine 0.01 mg/kg. The patient was then extubated and transferred to a recovery room.

The outcome assessor, who was an anesthesiologist not involved in the study, evaluated the outcomes.

#### Data to be collected

**Table:** 

1- Time of performance of the technique: the time from probe insertion until the visualization of local anesthetic spread
2. Time of onset of sensory block: the time between the injection of the drugs and the loss of pin prick sensation which was assessed every 5 min for 30 min following the block
3. Total intraoperative fentanyl consumption, excluding the induction dose, measured in micrograms
4. Pain intensity was assessed using Visual Analogue Scale (VAS) scores [13]. A commonly used visual analog scale is a 10-cm line labeled with "worst pain imaginable" on the right border and "no pain" on the left border. The patient was instructed to mark along the line to represent the intensity of pain currently being experienced. VAS scores were assessed at 30 min, 2 h, 4 h, 6 h, 12 h, and 24 h postoperatively. An intravenous increment of 15 mg nalbuphine (rescue analgesic) was given if VAS ≥ 4. 1 g of intravenous paracetamol was given every 6 h as a postoperative pain relief regimen, not exceeding 4 g in 24 h
5. The time to the first call for rescue analgesia (nalbuphine), which is the time between the end of surgery and the first report of postoperative pain, was recorded
6. The total amount of nalbuphine given to each patient during the first 24 h of the postoperative period was recorded
7. Any postoperative complications, such as nausea and vomiting, hypotension, bradycardia, phrenic nerve paresis, or any other complication, were noted

### Sample size

The primary outcome of the present study was the time to the first call for rescue analgesia (nalbuphine). We conducted a pilot study on seven patients from each group to estimate the sample size. The mean ± SD of the first time to call for rescue analgesia in the SC group was 9.28 ± 1.25 h, and 10.14 ± 1.07 h in the ES group, with 80% study power, α error of 0.05, and Beta error of 0.2. The number of patients in each group was 29. The soft online Open Epi program (https://www.openepi.com/SampleSize/SSCohort.htm) was used to calculate the sample.

size.

### Statistical analysis

IBM SPSS Statistics for Windows, Armonk, Version 23.0, NY: IBM Corp. 2015, was utilized for data collection and analysis. Quantitative data were presented as means and standard deviations (SD), or medians and ranges. Qualitative data were presented as numbers and percentages [N (%)]. A t-test was used to compare between the two groups of quantitative variables or Mann–Whitney if appropriate. The percentage of categorical variables was compared using the Chi-square test or Fisher exact test. All tests were two-sided. A *p*-value < 0.05 was considered statistically significant.

## Results

**Fig. 3:**
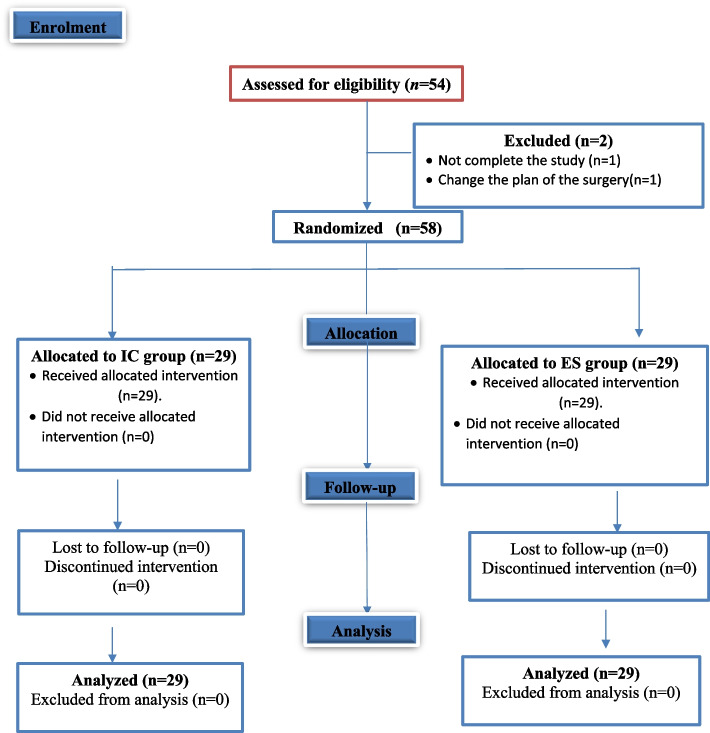
CONSORT diagram of the study

A total of 60 patients were enrolled in the present study. Two patients were excluded from the study—one patient did not complete the study and the surgery plan was changed for the other patient. The remaining 58 patients were randomly divided into two equal groups of 29 each (Fig. 3).

The characteristics of the patients and the duration of surgery were similar between the two groups (Table 1).

**Table 1:** Patients’ characters between the studied groups

Variables	Group SC *N* = 29	Group ES *N* = 29	t	*p*
**Sex** n (%)			0.069^c^	0.792
Females	14(48.3%)	13(44.8%)		
Males	15(51.7%)	16(55.2%)		
**Age (years)**	42.86 ± 9.02	44.62 ± 8.34	0.771	0.444
**BMI (Kg/m**^**2**^**)**	30.68 ± 2.3	31.24 ± 2.44	0.885	0.380
**ASA PS** n (%)				
I	14(48.3%)	11(37.9%)	0.633	0.246
II	15(51.7%)	18(62.1%)		
**Duration of operation (min.)**	97.1 ± 9.33	96.75 ± 7.85	0.152	0.880

Data were expressed as number and percent, or Mean ± SD [SD = standard deviation, t: student’s t test, χ 2 Chi-square test]

*ASA PS* The American Society of Anesthesiologists Physical Status, *BMI* Body Mass Index

^c^*P* ≥ 0.05 was considered not significant

The performance time was significantly shorter in the IC group (7.37 ± 1.2 min) compared to the ES group (14.21 ± 1.84 min) (*p* = 0.0001). There was also a significantly faster onset of sensory block in the IC group (15.62 ± 1.66 min) compared to the ES group (25.1 ± 4.06 min) (*p* = 0.0001) (Table 2).

**Table 2:** The analgesic properties of the block between the studied groups

Variables	Group SC *N* = 29	Group ES *N* = 29	t	*p*
Performance time (min)	7.37 ± 1.2	14.21 ± 1.84	16.708	0.0001*
Onset of sensory block(min)	15.62 ± 1.66	25.1 ± 4.06	11.633	0.0001*
The time to first call nalbuphine (h)	7.31 ± 1.34	11.10 ± 1.82	9.042	0.0001*
Total nalbuphine consumption (mg)	33.1 ± 10.13	22.76 ± 8.62	4.190	0.0001*

Data were expressed as Mean ± SD [SD = standard deviation, t: student’s t test

^*^P < 0.05 was considered significant, (Mann Whitney U test)

No patients required an additional dose of fentanyl beyond the initial dose in both groups.

**Fig. 4:**
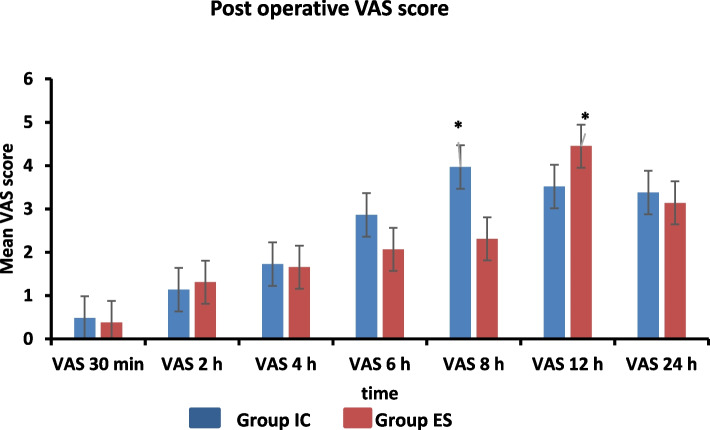
Bar chart with mean ± standard deviation for Visual Analogue scale (VAS) score between studied groups at the measured point time 24 h post-operative(*significant)

The average postoperative VAS scores were similar between the two groups at the measured time points (*P* ≥ 0.05), except at 8 h where the IC group showed significantly higher mean VAS scores compared to the ES group (*p* = 0.0001). At 12 h, the VAS scores were significantly higher in the ES group compared to the IC group (*p* = 0.0001) (Fig. 4).

The time to first request for rescue analgesia was significantly shorter in the IC group (7.31 ± 1.34 h) compared to the ES group (11.10 ± 1.82 h) (*p* = 0.0001). Additionally, the total nalbuphine consumption was significantly higher in the IC group (33.1 ± 10.13 mg) compared to the ES group (22.76 ± 8.62 mg) (*p* = 0.0001) (Table 2).

The total number of patients who experienced postoperative complications such as nausea, vomiting, bradycardia, hypotension, phrenic paresis, and Horner's syndrome was similar between the two groups (*P* ≥ 0.05) (Table 3).

**Table 3:** Postoperative complications between the studied groups

Variables	Group CS *N* = 29	Group ES *N* = 29	^f^*P*
Nausea	3 (10.3%)	5 (17.2%)	0.706
Vomiting	2 (6.9%)	2 (6.9%)	-
Bradycardia	4 (13.8%)	3 (10.3%)	0.99
Hypotension	4 (13.8%)	3 (10.3%)	0.99
Phrenic paresis	1 (3.4%)	0 (0.0%)	0.99
Horner’ syndrome	1 (3.4%)	0 (0.0%)	0.99

Data were expressed as number and percent, f = fisher Exact test

*P* ≥ 0.05 was considered not significant

## Discussion

The findings of the current study showed that ultrasound-guided bilateral intermediate cervical block did not provide longer postoperative analgesia compared to ultrasound-guided bilateral erector spinae block among patients scheduled for anterior cervical spine surgery. However, the procedure took less time to be performed and the sensory block started earlier with similar complications.

To date, no study has been found comparing intermediate cervical block and cervical erector spinae block in patients undergoing anterior cervical spine surgery.

### Anesthetic technique [5, 14–19]

Performance time and onset time were shorter in the IC group and comparable with published data by Vloka, Hipskind [5, 14, 15]. No repetitive opioid doses had to be administered intraoperatively, which we interpret as sufficient regional anesthesia during surgery (duration maximum 90 min) [16–19].

### Postoperative analgesia [11, 12, 20–26]

Reducing pain and preventing chronic shoulder pain following cervical spine decompression surgery are necessary to improve functional outcomes [20]. In the present study, the time to first request for rescue analgesia was significantly shorter in the IC group compared to the ES group. Additionally, the total nalbuphine consumption was significantly higher in the IC group compared to the ES group.

Intermediate and superficial cervical plexus blocks only address terminal sensory branches of the ventral rami of the C2-C4 spinal nerves (dermatomes). They do not block the motor branches (myotomes) of the ventral rami C1-4 or the dorsal rami that innervate the posterior neck muscles (myotomes) [11].

Moreover, the cervical part of the erector spinae muscle consists of the longissimus cervicis, semispinalis cervicis, and iliocostalis cervicis, which insert into the transverse process of C2 to C6 and extend from the thoracic to cervical region. Therefore, targeting C7 or C6 with the local anesthetic injection was effective in relieving shoulder pain, as demonstrated by other authors [12, 21–23]. This explains the superior postoperative analgesia provided by the erector spinae plane block compared to the intermediate cervical plexus block in the present study.

In agreement with the current study, Elmaddawy et al. [24] and Kannan et al. [25] found that the time to first request for pethidine in ultrasound-guided superficial cervical plexus block receiving plain bupivacaine was approximately 7 h postoperative in patients undergoing thyroid surgery. However, Kendall et al. [26] in their meta-analysis reported that patients undergoing orthopedic or spine surgeries experienced pain relief for up to 12 h postoperative when comparing erector spinae block with a control group.

### Potential complications [3, 11, 21, 23, 27, 28]

In the present study, the total number of patients who developed postoperative complications such as nausea, vomiting, bradycardia, hypotension, phrenic paresis, and Horner's syndrome was comparable between the two groups.

It is still controversial, if the prevertebral fascia is an effective barrier for injected local anesthetics. In this case the intermediate cervical plexus block would be a phrenic nerve sparing technique [11, 21].

It is likely that the use of lower concentrations and smaller volumes of local anesthetics minimizes the spread under the prevertebral fascia. In experienced hands bilateral intermediate block is considered a safe analgesic technique [29, 30]. In this study, we utilized a small volume and lower concentration of bupivacaine, and we included healthy volunteers classified as ASA PS I-II.

The spread of local anesthetic from the erector spinae plane to the epidural or paravertebral space depends above all on the volume of injected local anesthetic. Potential complications include circulatory changes (hypotension), unintended motor blockades and possible systemic toxicity at high LA doses. In adults, it is considered safe to use a local anesthetic volume of 20 to 30 ml [3, 23, 27, 28]. This corresponds to the volumes used in our study.

### Limitations

The cervical erector spinae plane block is a recently studied regional technique. However, there have been few studies conducted in the area of the current investigation. It is recommended to conduct further research to determine the optimal local anesthetic volumes and concentrations.

## Conclusion

For patients undergoing anterior cervical spine surgery, the intermediate cervical plexus block does not provide better postoperative regional analgesia compared to the cervical erector spinae block. Performance time and onset time were shorter in the IC group, whereas nalbuphine consumption was lower in the ES group.

## Data Availability

The data sets during this study are available from the corresponding author on reasonable request.

## Abbreviations

SC: Superficial cervical plexus
ES: Erector spinae
ASA: American Society of Anesthesiologists
BMI: Body mass index
VAS: Visual analogue scale
IV: Intravenous
